# Comprehension of informed consent and voluntary participation in registration cohorts for phase IIb HIV vaccine trial in Dar Es Salaam, Tanzania: a qualitative descriptive study

**DOI:** 10.1186/s12910-024-01033-z

**Published:** 2024-03-13

**Authors:** Masunga K. Iseselo, Edith A. M. Tarimo

**Affiliations:** 1 Department of Clinical Nursing, Muhimbili University of Health and Allied Sciences, Dar es Salaam, Tanzania; 2 Department of Nursing Management, Muhimbili University of Health and Allied Sciences, Dar es Salaam, Tanzania

**Keywords:** Informed consent, Clinical trials, Volunteers, HIV clinical trials

## Abstract

**Background:**

Informed consent as stipulated in regulatory human research guidelines requires volunteers to be well-informed about what will happen to them in a trial. However, researchers may be faced with the challenge of how to ensure that a volunteer agreeing to take part in a clinical trial is truly informed. This study aimed to find out volunteers’ comprehension of informed consent and voluntary participation in Human Immunodeficiency Virus (HIV) clinical trials during the registration cohort.

**Methods:**

We conducted a qualitative study among volunteers who were enrolled in the registration cohort of HIV clinical trials in Dar es Salaam, Tanzania. A purposive sampling strategy was used to obtain twenty study participants. The data were collected between June and September 2020 using a semi-structured interview guide. In-depth interviews were used to collect the data to obtain deep insights of the individual study participants on the comprehension of informed consent and participation in the clinical trial. A thematic analysis approach was used to analyze the data. Themes and subthemes were supported by the quotes from the participants.

**Results:**

Volunteers described comprehension of informed consent from different perspectives. They reported that various components of the informed consent such as study procedure, confidentiality, risk and benefits were grasped during engagement meetings. Furthermore, the volunteers’ decision to participate in the registration cohort was voluntary. However, trial aspects such as health insurance, free condoms, and medical checkups could have indirectly influenced their reluctance to withdraw from the study.

**Conclusion:**

Engagement meetings may increase the comprehension of informed consent among potential participants for HIV clinical trials. However, trial incentives may influence participation, and thus future research should focus on the challenges of giving incentives in the study. This will ensure comprehension and voluntary participation in the context of HIV clinical trials.

**Supplementary Information:**

The online version contains supplementary material available at 10.1186/s12910-024-01033-z.

## Background

In research involving human beings, informed consent is mandatory. There are several regulatory and ethical guidelines highlighting the need for prospective trial participants to understand the information provided during the informed consent process; these guidelines include the Nuremberg Code [1], the Council for International Organization of Medical Science (CIOMS) [2], Declaration of Helsinki [3] and Belmont Report [4]. However, the reality is that many prospective participants sign consent forms without fully understanding the contents of the forms [5–7]. A systematic review of studies conducted in low-income countries revealed that comprehension of informed consent is poor among study participants across Africa [8]. Thus, informed consent may be seriously compromised in these instances. Various factors influence the comprehension of informed consent among prospective participants. This includes language and medical terminology used, insufficient time allocation during the consent process, cultural/traditional reasons, and low education [9, 10].

The interest in informed consent comprehension in developing countries has been provoked by clinical trial experts questioning participants’ potential vulnerability and risk of research participation [11–13]. For example, participants may agree to participate in clinical trials to obtain some of the associated benefits of clinical trials without truly understanding the relevant facts and implications [14]. Furthermore, the influence of family and economic incentives may also affect comprehension and violate the principles of individual autonomy as applied in informed consent practice. Thus, educating individuals about the nature, purpose, and procedures of clinical research is ethically essential and likely related to the successful recruitment of trial participants [15]. A lack of knowledge about clinical trials has been documented in multiple communities [16, 17] indicating the need to provide information, dispel misinformation, and build trust in communities targeted for trials.

The process of obtaining informed consent continues to be a controversial issue in clinical and public health research carried out in low-income countries [18, 19]. For example, when assessing the willingness of volunteers to participate in Human Immunodeficiency Virus (HIV) Vaccine trials, it is also important to check the effectiveness of informed consent given to this group of people. The understanding of informed consent allows potential participants, based on their values, to decide which risks, benefits, and procedures are acceptable to them [20]. Importantly, some participants do not know what to expect and most are vulnerable to certain aspects. They are affected by information that is believed to be true in the community though this is not proven scientifically. These misperceptions include the side effects of vaccines on health in general, the presence of HIV within the vaccine, and the possibility of contracting or transmitting HIV as a consequence of participation [21]. In any HIV vaccine trial, these misperceptions need to be addressed appropriately to increase comprehension during the informed consent process.

In the HIV vaccine studies consent process, the guidelines state that informed consent must be obtained at all stages of the trials; i.e. screening, testing, administration of test product, repeat HIV testing, and any other examinations involved [22, 23]. It has been emphasized that prospective participants should also be informed of their relatively high-risk behaviour that can lead to HIV infection, the option to receive counselling and access to means for reducing this risk [24]. It is also important to let participants know that some participants in the trial may nonetheless become infected as a result of their high-risk behaviour and that only some of the participants will receive a test product, while others will receive a placebo [25]. These are all ethical issues that the participants need to be fully informed about, understand, and make voluntary decisions to participate without coercion or undue influence. Healthy volunteers may have altruistic motivations and actively assume some societal responsibilities [26–28]. Even in cases of extreme altruism, the right to withdraw is not thereby redundant since volunteers may genuinely change their minds or suffer effects that are unexpected or frightening [29, 30]. According to ethical principles, the decision to withdraw from the study for any reason provided by the participants needs to be respected [23]. These issues are important to be explored in detail among various study participants in any particular study.

Studies in different settings report poor comprehension of different components of informed consent in clinical trials [31–33]. However, the nature and method of presentation of information, demographic factors, and personal factors can positively or negatively affect the depth of comprehension of informed consent among research participants [34]. Modification of the provision of the informed consent process and rapid ethical assessment can improve comprehension among participants [35]. This is crucial for the effective implementation of clinical trials in low-resource settings.

In Tanzania, several HIV vaccine trials have been conducted and informed consent was provided and signed. However, anecdotal evidence shows that some participants who previously consented declined to continue with the trial, after consulting their significant others [36]. This raises questions about the quality and comprehension of informed consent provided during HIV vaccine trial preparation. In this regard, the question of to what extent the volunteers for the HIV vaccine trial comprehend the information in the informed consent remains unanswered. This study explored the understanding of informed consent and voluntary participation among participants in the registration cohort for the Phase IIb HIV Vaccine Trial in Dar es Salaam, Tanzania.

## Materials and methods

### Design

A qualitative descriptive study design was conducted to explore the comprehension of different aspects of informed consent. The choice of this design was appropriate due to the dynamic nature and ethical challenges of informed consent in clinical trials [37]. For this reason, it provided straightforward descriptions of understanding the topic under investigation and the nature of participation among the study participants [38]. Also, it recognised the subjective nature of the comprehension and thus presented the findings in a way that directly reflected or closely resembled the terminology used in the initial research question [39].

### Population and setting

The study was conducted among female sex workers (FSW) who were participating in the registration cohort for the HIV Pre-exposure prophylaxis and vaccine (PrEPVacc) trial in Dar es Salaam, Tanzania. PrEPVacc is a phase IIb HIV vaccine trial evaluating the efficacy of two HIV prophylactic vaccines with a second randomization to compare Descovy and Truvada as pre-exposure prophylaxis(PrEP) [40]. The cohort involved HIV-negative FSWs aged between 18 and 40 years who had consented to participate in the preparation phase of the trial. The trial site is located within the tertiary and referral health facility, Muhimbili National Hospital (MNH).

### Sampling methods and sample size

A purposive sampling strategy was used to obtain the participants. The procedure for screening, enrolment and follow-up visits is described elsewhere [41]. For this particular study, the process involved selecting participants who had rich information on the comprehension of informed consent that manifests sufficient intensity to illuminate the nature of success [42]. A research assistant who was conversant with the PrEPVacc registration cohort purposively identified the participants and invited them for interviews on a convenient day for them. This is because the first author (who conducted all the interviews) was an internal quality control team of the PrEPVacc study. The sampling process continued until information saturation was reached on the 20th participant, i.e. when no new information was elicited as far as the research question was concerned [43].

### Data collection

The data were collected between June and September 2020. This was one year after the participants had consented to participate in the registration cohort. A semi-structured interview guide was used during data collection. The guide was prepared in English and later translated into Kiswahili; the language commonly used by the participants. The main question focused on understanding the components of informed consent. i.e. information disclosure, comprehension, and voluntariness. The specific domains that were covered include the purpose of the research, risks, and benefits associated with participation, decision-making, and voluntariness of participation in the HIV vaccine study. These components were derived from the participant information sheet (PIS) and informed consent form that was used by the participant in the PrEPVacc registration cohort study. The PIS was adapted from the Ethics Review Committee of the World Health Organization (WHO) [44]. The interview guide was shared among experts in HIV vaccine trials for improvement of the content. The first author conducted all the interviews. All potential participants agreed to be audio-recorded. In-depth interview (IDI) was used to collect the data. We used IDI because we wanted to obtain deep insights from the individual study participants on the comprehension of informed consent in HIV vaccine trials. The interview took place at the research project site. This setting was used for the preparation of the PrEPVacc registration cohort during the scheduled visits. Thus, the participants were familiar with the environment. The interview was conducted in a well-lit room to enable the interviewer to capture the nonverbal cues from the participants during the interview. In addition, the setting was free from external voices. The interview guide which was developed by the researchers consisted of the following questions: (1) What did you consent to participate in? (2) What are the foreseen problems as a result of participation in that study? (3) In your perspective, what benefit do you expect to get as a result of participation in the study? (4) In your view, to what extent the information and samples you provide are kept confidential? 5)To what extent do you think participation in this study is voluntary? (**See Supplementary file**)

Each question was followed by specific probes to get a deeper understanding of the phenomenon. Follow-up questions were used as well for more clarification of the concept. The first three participants were used to pre-test the interview guide. This was important to determine if the questions in the interview guide were clear, understandable, and could be answered by the participants. Data from the three pre-test interviews helped to refine the interview guide by adding more probes and some question items were modified to increase clarity. The three pre-test interviews were not included in the current study sample. The interviewer (first author) who was an expert in qualitative research used most of the interviewing skills [45] which increased cooperation and a sense of acceptance among the participants. The interview continued until no information was obtained by adding new participants.

### Data analysis

The data analysis process started as soon as the first two interviews were completed. This helped to expand the probes according to the new information elicited from the participants. The audio-recorded data were transcribed verbatim by an experienced research assistant. The first author read each transcript to countercheck and ensure the accuracy of the information in the original audio data. Some corrections such as typing errors were made to enhance the clarity of the transcripts. Also, translations of the transcripts were made following the principles described by Chen & Boore [46]. The first author read and re-read all the transcripts carefully, line by line to be immersed in the data and get the first impression of the whole document. The second author read and re-read two transcripts and coded them manually on the margin of the paper. The first author used Nvivo 12 (computer-assisted data analysis software, CAQDAS) to organize and code all the transcripts. This increased ability to explore different possibilities of analysis and interpretation of the data [47].

**Fig. 1:**
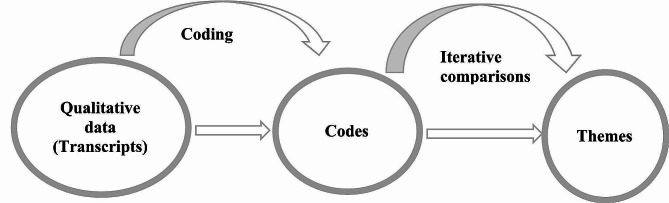
Process of Coding and Theme Formation

Thematic analysis was used to analyze the data. We used a hybrid approach that incorporated both the deductive and inductive approaches as outlined by Crabtree and Miller [48]. Deductive codes were derived from the main concepts as depicted in the research question and study title. We then created inductive codes by iterative reading and re-reading the transcripts to identify the most significant statements from the participants. This enabled the coding of the rest of the raw data which was not deductively captured in the previous phase. However, no major differences in categories formed between the coding approaches. We refined all the codes developed through both processes to ensure that all the patterns and meanings embedded in the participants’ text were included in the codes. Both authors shared the codes developed for comparison and discrepancies were discussed. We then compared the codes that we had created, identified patterns, and generated themes **(**Fig. 1**)**.

To ensure that the themes developed were accurate and representative of the data, we returned to the transcripts and compared the themes with the original data to confirm that we did not miss important information. We found that these themes were reflections of the textual data. In this process, some themes were split up into subthemes for explicit representation of the information. The final themes and subthemes were reported and supported with important quotes from the participants.

### Ethics approval and consent to participate

#### Ethical approval

was obtained from the Institution Review Board of Muhimbili University of Health and Allied Sciences with Ref. No. 282/298/01.C. Participants were well informed about the confidentiality of the information they would provide and that all the audiotapes, transcripts, and written notes containing the participants’ information would be kept confidentially after the completion of the study. Participants were informed of their right to withdraw or discontinue the study at any time they wished to. Written informed consent was obtained from all participants before the interview sessions. All methods were performed in accordance with the relevant guidelines and regulations as stated in the Declaration of Helsinki.

## Findings

### Socio-demographic characteristics

Twenty [20] participants provided information in this study. The mean age of participants was 27.0 years with a standard deviation (± SD) of 6.3. About two-thirds (13, 65%) had attained primary education and more than half (16, 80%) had experience in sex work between 1 and 10 years. The majority (16, 80%) of participants were mothers of at least one child **(**Table 1).

**Table 1:** Demographic characteristics of participants

Participants	Education level	Sex work experience (Years)	Number of children
Age (mean = 27.0, SD = 6.3) years			
1	College	2	3
2	Secondary	15	2
3	Primary	29	2
4	Secondary	5	1
5	Primary	5	1
6	Primary	3	1
7	Form 2	2	2
8	Primary	15	2
9	Secondary	15	1
10	Primary	3	1
11	Primary	2	1
12	Primary	6	1
13	Primary	15	1
14	Primary	13	2
15	Primary	7	2
16	Secondary	8	2
17	Primary	10	2
18	Primary	6	0
19	College	4	0
20	Primary	5	2

### Themes and subthemes

Participants’ understanding of various aspects of the research is described under the two themes namely Embracing study knowledge through informed consent and Consequences of Compliance to informed consent. For embracing study knowledge through informed consent; understanding of trial aim and duration, procedure and expectation as well as risks and benefits of research participation are described in detail. The consequences of compliance to informed consent honoured privacy and confidentiality, the nature of voluntary participation and the effects of incentives are elaborated in detail. Also, pitfalls of the right to withdraw from the study are described under this theme **(**Table 2**)**.

**Table 2:** Themes and Subthemes

No	Themes	Subthemes
1	Embracing Study Knowledge Through Informed Consent	1. Understanding the trial aim and duration
		2. Understanding of trial procedures and expectation
		3. Understanding the risks and benefits of trial participation
2	Consequences of Compliance with Informed Consent	1. Honoured Privacy and Confidentiality
		2. Nature of voluntary participation
		3. Effect of incentives and financial compensation
		4. Pitfalls of the right to withdraw from the study

## Theme 1: embracing study knowledge through informed consent

### Understanding the trial aim and duration

Many participants expressed the view that the study aimed to help those involved in commercial sex work, giving them protection against HIV infection. They described the study as an excellent project to reduce the high number of HIV infections among female sex workers. Also, the participants expressed interest in taking care of themselves until the vaccination became available and believed the project would eradicate HIV infection in the country. One participant stated:

*“The study itself aims to reduce or completely eradicate HIV infection. That is, by providing vaccination we should not get infected; so, the infected person can continue to use the medication, and the non-infected person may not get infected at all”* (P.No.08).

Other participants reported that the main goal of the research project is to develop an HIV vaccine that will help in the prevention of new HIV infections among the high-risk population. One of the participants said:

*“Its main goal is to make vaccines available. Thus, once a vaccine is available it can eradicate HIV infection, if possible”* (P. No.04*)*.

Some participants were not aware of the study duration. They recollected only the number of scheduled visits that were supposed to be attended. Even though some of them were not conversant with the exact number of visits that could be correlated with the study duration as stated below:

This study has taken… [hesitates], as I am here in the ninth visit. So, there are still about four visits to complete the number of scheduled visits. For me, I started last year, the month of … (she tries to think), Let’s say we are in the second year of research. So here we are at the end of the study” (P.No.08).

Regarding the trial duration, participants were aware that they were taking part in an HIV vaccine trial and were informed about the trial’s duration from the start. However, they did not consider as an important aspect to be comprehended as narrated below:

I still do not know how many years the study will take. You see, because the vaccine has not yet been found, we are still in the study and I think it will probably take maybe even… [Tried to remember but failed to get the answer]. We will participate until the vaccine becomes available” (P.No.13).

Although some participants reported the research to take two years or more, they envisioned that, once the research had started, it would be sustainable as far as the trial participants would be available to participate during the research period.

### Understanding of trial procedures and expectation

Many participants expressed that the most important procedure they consented to was attending the scheduled visits and getting checked for health status. They said they were testing for HIV because the project required a person who was not infected with HIV.

*“I was told I had to get tested because once infected, you cannot get into the study. Once infected, you can no longer get vaccinated. So, the vaccine does not require a person with a serious illness, such as hepatitis, syphilis, or hypertension, because of an unknown vaccine’s reaction (P*.No.07).

They also asserted that every three months there were long and short visits. When they come for a long visit, they are tested for hepatitis, HIV, and pregnancy. When it comes to short-term visits they only tested for HIV. They stated that the pregnancy test was one of the important tests at that time because the project aimed to exclude pregnant women for safety purposes, particularly for the unborn baby. They also reported testing for STIs because they faced so many challenges as sex workers. One participant expressed:

*“As I understand we are being tested for pregnancy because you can get pregnant any time. If enrolled in this study, at the end of the day you can get a problem such as miscarriage or a baby born with abnormalities. That is why they said, vaccination is not recommended for pregnant women*, (P.No.14)

However, some participants reported that they did not understand the details of various tests as described below:

“*They did not tell me the reasons for testing. But they told me you are testing just to look after your health”* (P.No.11).

Participants described that even the participation in the actual vaccine trial would be determined by those who were attending regular visits. They reasoned that they would not be included in the vaccination if they had poor attendance to the scheduled visits. They also added that regular visits to the clinic were beneficial as they were given education about research and vaccination.

### Understanding the risks and benefits of trial participation

Participants verbalized that providing blood samples was not a problem. They only stated feeling some pains due to the injection or mild headache after donating the blood. These events were described as a minor side effect that human beings could tolerate and also can be found in any other health facility providing such services as stated by one participant:

*“We saw the effects in the early stages of participation when coming to give the blood sample, it hurts. That is the only minor harm or injury. But for effects like feeling dizziness, nausea after donating blood sample, I don’t think it can be considered a major risk for participating in this trial, it depends on the person and how he/she takes it”* (P. No. 13).

Many participants reported fear of HIV test results because of risky sexual behaviour undertaken during sex work. They said, sometimes they get clients who do not want to use condoms. Although they were counselled to use protective methods during sexual activities with their clients, they described it to be difficult to implement due to the desire to get a high amount of money when they practice sex without condoms as expressed below:

*“I must be scared because I am in the sex business. Someone comes with his load* [a large amount of money], *he tells you I don’t want to wear* [use a condom], *I go* [have sex] *with him dry [*condomless], *so I go without a condom”* (P.No. 09).

Concerning the benefit of participation, a few participants expressed increased confidence in their sex work. They said the vaccine derived from participation in the study would help in the prevention of HIV infection from their customers and therefore no longer be afraid of contracting HIV. They stated that when meeting with a male customer /client who engages in sex without a condom, they would not be worried anymore like in the beginning as expressed by one of the participants:

*“The first benefit is that I will be safe, I will have sex without stress, and I will not get HIV infection. Since I will have received this vaccine, I will be safe, I will have sex with confidence. If I go with him dry* [without a condom] *my money will increase. I will make important goals of running my family”* (P. No 14).

Participants stated that getting checked on their health status was one of the benefits obtained from participation in the study because they had hardly been tested before. They expressed that, although HIV testing services were freely offered in all hospitals, testing for other STIs such as gonorrhoea and syphilis had cost implications that prevented them from checking their health status as expressed below:

*“You know testing for HIV is free at the hospital. But to test for gonorrhoea, syphilis must be paid for But when I come here, I get tested for all STIs to know my health in general*” (P.No.17).

They added that participating in the study had increased their morale of knowing their health status. They said that having someone to remind and encourage was an important aspect for them that they had not experienced before joining the study as revealed below:

*“If someone has not persuaded you, you cannot go straight to the test for HIV. But if somebody comes and persuades you that if we do this, there is more relief. That is why it was hard on me to check my health status at the facility*” (P.No.20).

Some participants also asserted that participation in the study was a chance to increase their social network particularly when meeting with other participants from different places in Dar es Salaam. They said that the scheduled visits would help in coming out and having fun and exchanging sex work information with others. They also expressed that the collective benefit of the effective vaccine that could have resulted from their participative efforts would free people from the risk of HIV infection.

“*In the future, the benefits that will come out of this* [study]*are not just for me, it will benefit the whole community. Because once you have completed the vaccine trial, the vaccine will be available for everyone” (P. No*.17).

They also added that if the vaccine were available and they were lucky enough to get vaccinated, it would be a benefit to them because they would be free to do their business.

## Theme 2: consequences of compliance to informed consent

### Honoured privacy and confidentiality

Many participants expressed that they were informed about the sample collection and that the information they would provide during the study period would be kept confidential. They further elaborated that, the use of numbers instead of names was a very good strategy that increased the sense of trust that their information remained non-identifiable as expressed by one of the participants below:

“What I like is that they do not call names, it is just numbers. So, I have found there is a lot of secrecy that’s why I am not worried. I think my information will be confidential” (P. No. 01).

The use of computers in data entry and preservation of participants’ information was similarly considered an essential component for maintaining privacy and confidentiality. Participants reported that electronic data handling enhanced trust among the research participants and the research team as stated below:

*“They use computers. Names are not entered into the computer, they put our numbers that you see. I like it because I only know that number, but no one else knows*” (P.No.13).

A few participants also expressed that, the research team was trustworthy in that they had counselling skills that helped them to understand how their information would be handled and processed. They said, their work would be jeopardized if the information would not be kept confidential. They verbalized that the research team helped to allay participants’ anxiety as narrated by one of the participants:

*“When I came here, I told them, I am a sex worker, how do you assure my privacy? These sisters* [counsellors and nurses] *reassured me, …until now, I observe privacy. This means no one knows what I am coming here for”* (P. No. 8).

Participants were also confident that the data and information they provided could not leak because they believed those who work in health facilities, to a very large extent, have taken oaths. They commented that it was not possible to be a doctor or a nurse without taking an oath as part of their ethical practice as expressed below:

*“I don’t think my information can leak because I believe those who work here have taken oaths. This is because you cannot be a doctor or a nurse without taking an oath*” (P. No. 13).

Participants praised the way laboratory results were handled. They insisted that it was the right way of giving test results without the presence of other people around. They argued that the room that they used for testing was well furnished which contributed to the high sense of privacy. This maintained confidentiality from data collection to giving results in such a way that no one else could hear or know the test results.

*“We were told, for example, if you have been tested and found to be HIV positive, no one will know besides your doctor who tested you or gave your test results*” (P.No. 12).

### Nature of voluntary participation

Many participants expressed that participation in the registration cohort for HIV vaccine efficacy trials was voluntary and that they did not feel pressured to participate. They verbalized that they had the freedom of choice whether to join the study or not. In addition, participants described that their decision to participate in the study was not influenced by other external forces. In this case, participants revealed the ability to make their own decisions coupled with the absence of coercion for participation; illustrating the overriding importance of free will and individual choice as described below:

From that first day, we were not told if you sign you will be given money or what. As I saw this, something will help me, even if I am not there, it will help others. Because I participated even if there were no such things, I would participate because I had free will. (P.No. 02)

Participants commented that participation in the study was voluntary to a certain extent because when they started, everyone was given a form to fill in. They said that each person had to read and understand those forms before signing. They also augmented that there were no payments for participation in the study and therefore needed to volunteer as expressed below:

*“I wanted to participate through my own free will. I was not forced by anyone. I decided to take part in the study after understanding the purpose of the study.*” (P.No.19).

Some participants went further by emphasizing the importance of meeting the criteria and eligibility of the study rather than the voluntariness alone to participate in the vaccine trial. They said that even if participation was their choice, they should meet the study eligibility criteria as expressed below:

*“I think meeting the criteria is the priority. You may want to participate voluntarily, but if you do not meet the criteria, you will not be a participant. Or if you meet the criteria but you are forced, you will not be a participant. So, if you are willing and you meet the criteria you can be a participant”* (P.No.13).

### Effect of incentives and financial compensation

Many participants pointed out that financial compensation such as transport reimbursement and refreshments that were offered to them could not influence their decision to participate in the prospective vaccine trial. They described that those who expected money as a criterion to participate were not willing and left the registration cohort. This was revealed by one of the participants as shown below:

*“I am not the type of person to be given those things* (money) *to participate*… *so you get the reputation of saying ‘I am going to that place because I am given the money. Not really. First of all, the amount of money I get here is smaller than what I get from my work”. So, participation in this study is not influenced by the money”* (P.No.16).

Some participants described mixed feelings on the role of incentives as a propellant of participation in the study. They verbalized that some services that they were informed to receive as part of the study might have contributed to the consent to participate in the study. They said that the financial challenges coupled with difficulties in obtaining menstrual hygiene materials could have indirectly contributed to continuing participating in the study as stated by one of the participants below:

Yes, they may have attracted me to continue being in the research, because we have things that we are missing. For example, as for the pad or tampons needs, if they give me a pad, when it comes to my menstruation, it can help to move on. In short, such things can make a person attracted even if has no intention (P. No. 02).

The participants expressed that they were given transport reimbursements because of the time they wasted during the clinic activities. Due to the many clinical procedures that each participant had to undergo, for them, it was the right thing to be given such support that could compensate particularly for transport. They stated that such an offer could not be regarded as motivation for them to participate in the study.

### Pitfalls of the right to withdraw from the study

An important aspect of voluntary participation in research is the capacity to withdraw from an ongoing study. In this aspect, participants narrated that they had the right to discontinue the study at any time they felt to do so. However, they said they had to explain first the reason for the withdrawal from the study. They added that if they had clear reasons for quitting, the study team would not force the participant to stay in.

*“If I want to stop participating, I have the right to report to the study team because I agreed and signed the consent form.”* (P.No.07).

Concerning the consequences of withdrawing from the study, participants revealed that they did not think they would be affected in any way. They asserted that there was no penalty for the utilization of usual health services for such withdrawal except that some research incentives would be lost. They mentioned incentives such as health insurance, free condoms, and medical checkups would be lost because they were directly linked to participation in the study. They emphasized condoms were important for their daily commercial sex work.

*“If I give up, some of the benefits I get will be affected. Because there will be no one to advise me anymore, I will be going as I am. There will be no one to listen to me, no one to educate me, at the end of the day I will be lost”* (P.No.17).

Some participants further expressed that one can stop participating in the study if she gets pregnant or infected and this would be the right of the study team or investigators to exclude from the trial. They added that the trials had several criteria which every participant should have met to be enrolled in the study or continue to participate in the study as asserted by the participant herein:

*“You can stop if you are pregnant or infected. It is the right of the project owners to exclude you from the project, and you have the right to leave the project because they do not want you to get pregnant or infected. So, you cannot be forced to stay. Just information is the key”* (P.No.08).

## Discussion

This study aimed to explore the understanding of informed consent and voluntary participation in the registration cohort for the Phase IIb HIV Vaccine Trial. The participants described comprehension of informed consent according to their level of understanding. The understanding of the purpose, risks and benefits of participation, trial procedures and confidentiality fairly emerged in the current study. However, the nature of the participants’ occupations might have influenced their voluntary participation in the study. Since the main purpose of the vaccine trials was to develop an HIV vaccine that would help in the prevention of new HIV infections among the high-risk population, participants’ understanding of the purpose is coupled with their desire for HIV risk reduction as a consequence of commercial sex work.

In conjunction with the information disclosed by the trial staff about the study purpose during consenting, the participants have shown adequate comprehension similar to the study reported in Romania [49]. On the other hand, the duration of the trials was understood by the participants in terms of the scheduled visits and total registration period preparation. This indicates the information provided by the staff team is congruent with the requirements of the ethical guidelines [1–3] and as per the study protocol. Notwithstanding this understanding of the study duration, it should be borne in mind that our finding is based on the registration cohort only. The actual understanding of the trial duration might have been affected by the continued engagement meetings between the study participants and the trial staff.

The expression that every three months there were long and short visits indicates that the participants understood the planned trial procedure. This is attributed to the regular engagement meetings conducted to enhance participants’ comprehension. Engagement meetings are one of the strategies reported to improve comprehension of informed consent among the trial participants [50, 51]. The understanding of specific tests such as HIV and pregnancy during the specified visits was the cornerstone of the registration cohort. It was also crucial for establishing the incidence among the potential trial volunteers as reported in the previous study [41]. In the current study, an engagement meeting is important because participation in the actual vaccine trial is determined by proper attendance and adherence to the stipulated procedures. The good understanding of the study procedure in our study demonstrates that participants comprehended the information provided by the trial team. This is consistent with other studies reported elsewhere [5, 52]. Furthermore, regular visits to the clinic and sample testing are beneficial procedures. This is because the health education provided by the study team helped to know their health status. These activities motivate participants to participate in clinical trials as reported in other studies [53, 54]. The pregnancy test as one of the procedures in the registration cohort was well understood by the participants. This is because the project aimed to exclude pregnant women for the safety of the unborn baby [55, 56]. This prerequisite condition increased their effort to understand the study procedure and what to expect during the study.

Concerning the risks and benefits of participation in the trials implies that the comprehension of risks in the current study might be attributable to the nature of the study. The risks explained in the consent form and during the consent process were discomfort during sample collection and embarrassment during physical examination. For the pain due to injection, while taking blood samples, the participants might not have perceived these as major ‘risks’ because they might have experienced these discomforts before, sometimes with more sophisticated procedures [57]. These events are minor side effects that human beings can tolerate. Our findings are consistent with other studies reported in the United States (US) where respondents minimized the risks of research participation [24, 58]. The reported fear of HIV test results in the current study is regarded as a risk for participating in the trials. This fear indicates that participants do not have regular HIV testing because of risky sexual behaviour undertaken during sex work. This is similar to other studies reported in China and Indonesia [59, 60]. The inconsistent use of condoms increases the fear of HIV testing due to the increased chance of HIV infection among the study population. The high-risk behaviour is motivated by the financial gain after condomless sex as reported in South Africa [61] and India [62]. It is worthwhile to note that the risk of minor discomfort and the known safety of the vaccines are well described in this study. However, one of the major risks is the notion that trial participation is somehow protective, which may lead to riskier behaviour and subsequent HIV infection. This is consistent with systematic reviews that found an increase in risky sexual behaviour following HIV prevention trials [63, 64].

The trust towards the research team in the confidentiality of information and data as reported in this study may be described based on ethical principles of respect for persons [65, 66]. The use of computers in data entry and preservation of participants’ information increases trust among study participants similar to other studies reported elsewhere [67–69]. The ability to handle participants’ information in the current study indicates that the project recruited a research team who are competent in education, experience and training as recommended in Good Clinical Practice (GCP) for clinical trials [70]. Additionally, the research team regularly received training in research ethics that enhanced their competencies in handling participants’ information and providing informed consent.

The voluntary participation as expressed by the study participant indicates that participants have altruistic motives and consciously assume certain responsibilities to the wider society as reported in other studies [26–28]. The intention for voluntary participation was not affected by any other undue influences such as financial reimbursement. This is contrary to studies in the US which reported that financial reimbursement increased willingness to participate in HIV studies [71, 72]. It is also worthwhile to note that meeting the eligibility criteria of the study is a prerequisite condition to participate in the HIV clinical trial [73] rather than the voluntariness alone as stated by the participants in our study.

The right to withdraw from participation in research is recognized in virtually all national and international guidelines for research on human subjects [1–3]. The concept also applies to biobank samples as reported by Helgesson and Johnsson [74]. Although the withdrawal right was described during the consenting process, participants in the current study understood this information in the light of the perceived benefit of participation including the free medical checkups. The importance of the inclusion of information about the right to withdraw in the consent form has also been emphasized and reported in other research findings [74, 75]. The reason that only pregnancy and HIV acquisition remove the participant from participating is linked to the understanding of the eligibility criteria of the proposed trial. This indicates that the study protocol and procedures were well-grasped by the study participants and the consequences of withdrawing from the study cannot be underestimated. Although, it is stipulated that no penalty for the utilization of usual health services for participants’ withdrawal [76], this study found that volunteers may not voluntarily leave the study due to the indirect benefits obtained from the study. This can be explained by the fact that our study sample was a high-risk and marginalized population; hence, have little access to HIV prevention services in other settings as a result of self-stigma [77]. This might have decreased the tendency of volunteers to withdraw from the study once they were recruited.

Several limitations can be described in this study. First, findings may not be transferred to other Tanzanian sites due to the differences in the goals and procedures of the study they were consenting to. Second, the translation of the participant’s excerpts from Kiswahili to English represents another possible limitation. However, a process of back-translation was implemented by experts such that the final statements represent the participants’ intended meaning. Third, in our study, we did not attempt to explore participants’ prior knowledge of participation in similar HIV prevention studies. Thus, we do not know if the comprehension of informed consent and participation might be influenced by prior experience in other studies.

Our study contributes to the literature on voluntary participation and comprehension of informed consent to participation in HIV prevention studies in similar settings in Tanzania. The importance of qualitative empirical research on applications of requirements for informed consent to HIV prevention studies will increase as more people in the community, particularly the high-risk groups volunteer to participate in such studies. Nevertheless, the nature and purpose of the studies and their associated risks and benefits vary in complexity and this can influence the process of communicating information during the consent conversation.

## Conclusion

Although the regulatory guidelines describe what the informed consent process should involve for individual participation, in practice the beliefs, values, trust and power dynamics during clinical trial implementation may influence the consent process in which the participants and the research team interact with each other and with their wider social networks. The informed consent process not only involves obtaining a signature from the participants but also continuous engagement between the research team and the volunteers throughout the clinical trial. However, trial procedures may influence participation, and thus future research should focus on the challenges of giving incentives in the study. This will ensure comprehension and voluntary participation in the context of HIV clinical trials. This will further our knowledge of comprehension and voluntary participation in the context of HIV clinical trials.

### Electronic supplementary material

Below is the link to the electronic supplementary material.

Supplementary Material 1

## Data Availability

The dataset supporting the conclusions of this article is included in the article.

## Abbreviations

CIOMS: Council for International Organization of Medical Science
CAQDAS: Computer-assisted data analysis software
GCP: Good Clinical Practice
HIV: Human Immunodeficiency Virus
IDI: In-depth interview
MNH: Muhimbili National Hospital
PIS: Participant information sheet
PrEP: Pre-exposure Prophylaxis
PrEPVacc: Pre-exposure prophylaxis and vaccine
WHO: World Health Organization
