# Supplementary material for: Comprehension of informed consent and voluntary participation in registration cohorts for phase IIb HIV vaccine trial in Dar Es Salaam, Tanzania: a qualitative descriptive study
Source: BMC Med Ethics. 2024 Mar 13;25:29. doi: 10.1186/s12910-024-01033-z (PMC10935914; doi:10.1186/s12910-024-01033-z)
Supplement: Supplementary file 1 — Supplementary Material 1 [file 12910_2024_1033_MOESM1_ESM.docx]

| **Interview guide for comprehension of informed consent for the participant in the PrEPVacc preparedness study** |
| --- |

1. **Self-introduction**
2. Can you describe to me in detail what type of research are you participating in? Probes: Who is involved; Who is the sponsor; How long will the study last? Please, tell me how were you selected? What made you selected among your colleagues/friends
3. What is the aim of this study? (Probe )
4. What do you think are the procedures for participating in this study? What are your views on the scheduled visits? What tests are compulsory to take when you are enrolled in this study? What are your views on the blood samples taken? (Probe)
5. What are the possible risks or discomforts associated with participation in this study? (Physical or psychological or social?). Can you tell me other risks that you wish to describe?
6. What will happen to the information collected in this study? How is the confidentiality of your information ensured in this study? Can you tell me who is allowed to see your medical records for this study? What will happen to the results of this study? (probe)
7. Describe the benefits that you are expecting as individuals and others as a result of this study? (Probe)
8. What are the compensations involved in this study? What is the purpose of reimbursement/compensation that you are receiving from this study? Can you tell me more about the adequacy of this compensation? (Probe)
9. Your participation in this study is voluntary. What would happen if you had refused to be in the study? Tell me your rights to withdraw from the study and give reasons (Probe)
10. Who can you contact if you have questions regarding the study and about your rights as a research participant? What are your views and suggestions on how to improve the conduct of this project (communication system with the study team, logistics, etc) (Probe)
11. Did you involve anyone in your decision to participate in this study? If yes, what is your relationship with that person, and why did you involve him/her (Probe) If no, what are the reasons?
12. Do you have anything else you want to discuss regarding this interview/study? (Probe)

**Thank you for participation**

**MWONGOZO WA MAHOJIANO**

1. **Utambulisho**
2. **Nieleze kwa undani ni aina gani ya utafiti unaoshiriki.** Ni watu gani wanahusika na utafiti huu? Mfadhili wa utafi huu ni nani? Utafiti huu utadumu kwa muda gani? Tafadhali niambie ulichaguliwaje? Kitu gani kilikufanya uchaguliwe wewe kati ya wenzio ? (Dadisi )
3. **Kusudi na lengo la utafiti huu ni nini?** (Dadisi)
4. **Unafikiri utaratibu wa kushiriki katika utafiti huu ni upi?** Nini maoni yako kuhusu mahudhurio? Vipimo gani muhimu vitafanyika/au vimekwisha fanyika kwenye utafiti huu? Una maoni gani kuhusu kuchukuliwa vipimo vya damu? (Dadisi)
5. **Nini hasara au athari unazoweza kupata kutokana na kushiriki katika utafiti huu?** (kimwili, kisaikolojia au kijamii?) Unafikiri kuna athari zingine ambazo ungependa kueleza? Kama ndiyo, athari hizo ni zipi? (Dadisi)
6. **Nini kitatokea kwenye taarifa ulizokwisha toa kwenye utafiti huu?** Umehakikishiwaje usiri wa taarifa zako katika utafiti huu? Ni nani anaweza kuona taarifa zako ulizotoa katika utafiti huu? Matokeo ya utafiti huu yatafanyiwa nini? (Dadisi)
7. **Nieleze faida za kushiriki kwenye utafiti huu; kwko wewe na kwa wengine** (Dadisi)
8. **Ni fidia ipi unapata kutokana na kushiriki kwenye utafiti huu?** Nini malengo na madhumuni ya kulipwa fidia? Unaweza kuniambia zaidi kuhusu kutosheleza au kutokutosheleza kwa fidia hiyo? (Dadisi)
9. **Ushiriki wako kwenye utafiti huu ni wa hiari. Nini kingetokea endapo ungekataa kushiriki kwenye utafiti huu?** Niambie kama una haki ya kujitoa na sababu zake (Dadisi)
10. **Ni nani unaweza kumuona ukiwa na swali kuhusu utafiti huu au haki zako kama mshiriki wa utafiti huu?** Nini maoni na mapedekezo yako kuhusu kuboresha mwenendo wa utafiti huu? (hasa kwenye mfumo wa mawasiliano na timu ya watafiti?) (Dadisi)
11. **Ulimhusisha mtu yeyote katika uamuzi wako wa kushiriki katika utafiti huu?** Ikiwa ndiyo, uhusiano wako na yeye ni upi na ulimshirikisha namna gani? Kama hapana, toa sababu (Dadisi)
12. **Una kitu chochote kingine ungependa kuzungumza kuhusu mahojiano haya au utafiti huu unaoshiriki?**

**Asante kwa kushiriki**
